# Neuroblastoma and nephroblastoma: a radiological review

**DOI:** 10.1186/s40644-015-0040-6

**Published:** 2015-04-08

**Authors:** Maureen Dumba, Noorulhuda Jawad, Kieran McHugh

**Affiliations:** Department of Radiology, Great Ormond Street Hospital for Children, Great Ormond Street, London, WC1N 3JH UK

**Keywords:** Neuroblastoma, Nephroblastoma, Wilms tumour, Review, Staging, Imaging

## Abstract

Neuroblastoma (NBL) is the most common extra-cranial tumour in childhood. It can present as an abdominal mass, but is usually metastatic at diagnosis so the symptomatology can be varied. Nephroblastoma, also more commonly known as a Wilms tumour, is the commonest renal tumour in childhood and more typically presents as abdominal pathology with few constitutional symptoms, although rarely haematuria can be a presenting feature. The pathophysiology and clinical aspects of both tumours including associated risk factors and pathologies are discussed. Oncogenetics and chromosomal abnormalities are increasingly recognised as important prognostic indicators and their impact on initial management is considered. Imaging plays a pivotal role in terms of diagnosis and recent imaging advances mean that radiology has an increasingly crucial role in the management pathway. The use of image defined risk factors in neuroblastoma has begun to dramatically change how this tumour is characterised pre-operatively. The National Wilms Tumour Study Group have comprehensively staged Wilms tumours and this is reviewed as it impacts significantly on management. The use of contrast-enhanced MRI and diffusion-weighted sequences have further served to augment the information available to the clinical team during initial assessment of both neuroblastomas and Wilms tumours. The differences in management strategies are outlined. This paper therefore aims to provide a comprehensive update on these two common paediatric tumours with a particular emphasis on the current crucial role played by imaging.

## Introduction

Neuroblastoma (NBL) is the most common extra-cranial tumour in childhood. It can present as an abdominal mass, but it is often metastatic at diagnosis, so can manifest in a variety of other ways often in an unwell child. Nephroblastoma, also more commonly known as a Wilms tumour, is the commonest renal tumour in childhood and more typically presents as abdominal pathology in an otherwise asymptomatic patient. The natural histories and typical clinical courses of these common tumours are very different. This paper reviews the clinical aspects and imaging characteristics of both tumours, summarising the key differences to aid the paediatrician and radiologist in their diagnosis and management.

## Review

### Neuroblastoma

#### Background

Neuroblastoma (NBL) is the most common solid extra-cranial tumour in childhood [1]. NBL arises from primordial neural crest cells that form the sympathetic nervous system, occurring anywhere along the sympathetic nervous system chain [1,2]. Under the microscope, they are small, round, blue cells that are clustered in rosettes. They share similar cell characteristics on gross histological evaluation to other relatively common paediatric tumours such as Ewing’s sarcoma, primitive neuroectodermal tumours (PNETs), leukaemia, lymphoma and rhabdomyosarcoma [3].

Typically, NBLs occur in early childhood with up to 90% diagnosed by 6 years of age [4]. Incidence is slightly higher in Caucasians. The heterogeneity of the tumour, and its biological characteristics, mean the prognosis is highly variable at different ages. Some behave aggressively while others, typically in infancy, may spontaneously regress. This variability means survival rates also differ. Low to intermediate risk tumours tend to have a reasonably good prognosis (90% survival approximately) with high-risk tumours being much less favourable (40-50% survival) [5]. In 2002, approximately 15% of childhood cancer deaths were due to NBL [6].

#### Associations and risk factors

The vast majority of cases are sporadic. Approximately 1% are familial, displaying an autosomal dominant pattern of inheritance with incomplete penetrance [1,4]. There are a wide number of conditions that have been associated with NBL; neurofibromatosis type 1, Beckwith-Weidemann syndrome, Hirschsprung’s disease and DiGeorge syndrome are all described in the literature [6].

NBLs have a variable prognosis; tumour stage, patient age, tumour oncogenes and DNA content are all known to be implicated. The MYCN oncogene is responsible for providing the code used by proteins in tissue development. If this mutates, which can be signalled by abnormal amplification, cancerous cells can develop and the resulting mass is more resistant to treatment, thus it has a more unfavourable outcome [1]. This negative feature occasionally includes those children who otherwise have favourable tumour presentation features, for example 4S/MS disease and young age [1]. Tumours with MYCN amplification, whether localised or metastatic, are all categorised as high risk tumours in both North American Children's Oncology Group (COG) and European (SIOPEN) neuroblastoma studies. NBL with DNA that has a hyperdiploid structure appears to be less aggressive. This is thought to be secondary to a reduction in mitosis [1].

Other markers which can affect management include chromosomes and nerve receptors. Alterations in two chromosomes, namely a deletion on the short arm of chromosome 1 (1p) seen in around a quarter of NBLs, and deletion of chromosome 11q have a poorer prognosis [1]. Although 1p deletion is associated with MCYN amplification, 11q is not correlated and appears to have separate negative prognostic factors. TrkA is a neurotrophin receptor that may actually be associated with an improved prognosis.

#### Clinical features

Presenting features are diverse and very much dependent on the anatomical location of the tumour. Most commonly, NBLs are located within the adrenal gland, but can be found in sympathetic ganglia of the retroperitoneum, posterior mediastinum, neck or pelvis [1]. The organ of Zuckerkandl is a mass of neural crest tissue adjacent to the mid to distal abdominal aorta and it is another recognized site of disease.

Abdominal masses usually cause pain due to their mass effect, as well as abdominal distension [6]. They often grow to a large size before causing problems, so a palpable mass on presentation is common. Abdominal masses can also compress renal vessels resulting in hypertension being a presenting feature. Thoracic NBL may present with airway compromise, scoliosis or as an incidental finding on chest x-ray. The biology of thoracic NBL tends to be less aggressive than with abdominal disease and as such the prognosis tends to be more favourable.

Paraneoplastic syndromes may be associated with non-metastatic disease. One of these syndromes is opsomyoclonus, complicating 2-4% of presentations [1,6]. The other is the excessive production of vasoactive intestinal peptide (VIP) resulting in watery diarrhoea and failure to thrive [1]. In addition to local disease, metastatic disease complicates 50% of all presentations. Common sites of metastases include liver, lymph nodes and bone marrow [1]. NBLs can metastasize to the skull base and orbital floor resulting in periorbital ecchymoses and a so-called “raccoon eye” appearance [1,3].

#### Diagnosis

**Figure 1:**
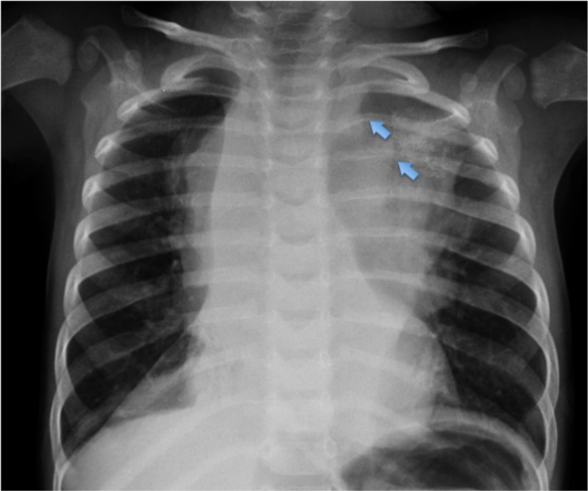
**Chest x-ray of 3 year old girl showing thoracic NBL. Note erosions of the posterior 3** ^**rd**^ **and 4** ^**th**^ **ribs indicating a posterior mediastinal mass.**

Plain films are non-specific for NBL and are largely unhelpful in the diagnostic pathway. Tumours of the chest and neck can be incidentally picked up on radiographs performed for other reasons [Figure 1]. Features that are suggestive of thoracic disease include abnormalities of the normal silhouettes typically seen on chest x-rays. The right and left paraspinal lines are where the lung or pleura interacts with the mediastinal soft tissues. In children, the paraspinal lines are less frequently observed than in adults due to less mediastinal fat and no aortic ectasia. Thickening and irregularity of these lines, in particular the right paraspinal line which is not normally seen in healthy children, can indicate the presence of increased mediastinal soft tissue and this warrants further investigation [7].

Ultrasound (US) is often the first line investigation in paediatrics, particularly for those presenting with an abdominal mass. NBLs appear as solid, heterogeneous masses with calcification, but are rarely cystic on US [8]. For surgical planning and risk stratification, more detailed cross-sectional imaging is necessary.

**Figure 2:**
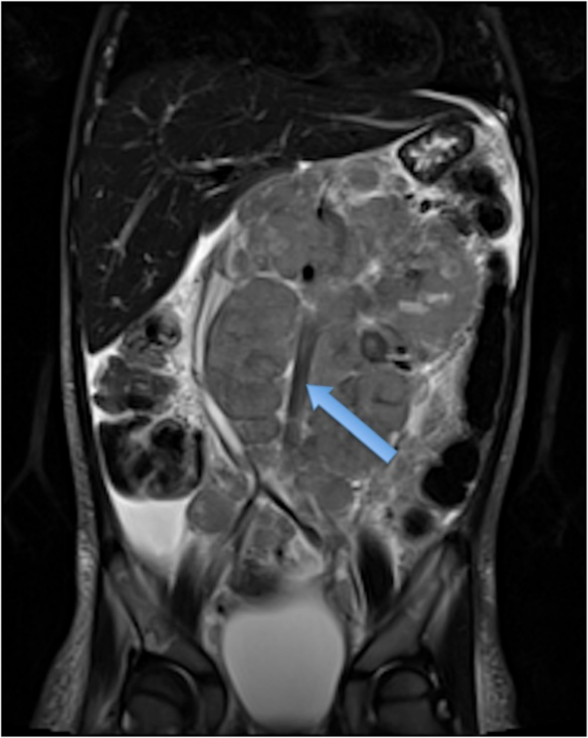
**Coronal T2 MR of a 3 year old boy with extensive abdominal NBL that crosses the midline and is here seen to encase the aorta (blue arrow).**

**Figure 3:**
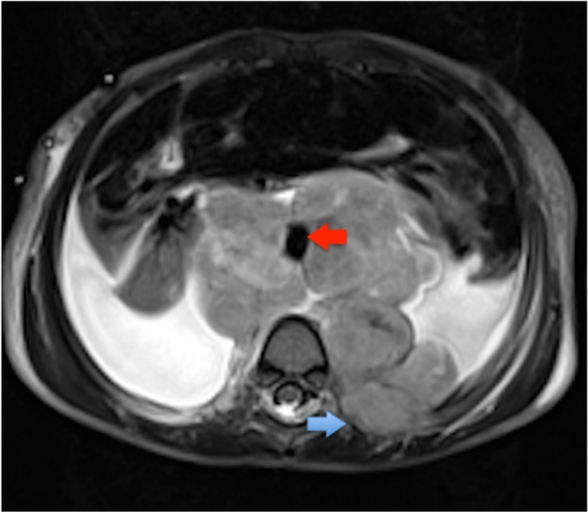
**Axial T2 MR of 2 year old girl showing NBL with rib invasion (blue arrow), anterior aortic displacement and encasement (red arrow) and bilateral pleural effusions.**

**Figure 4:**
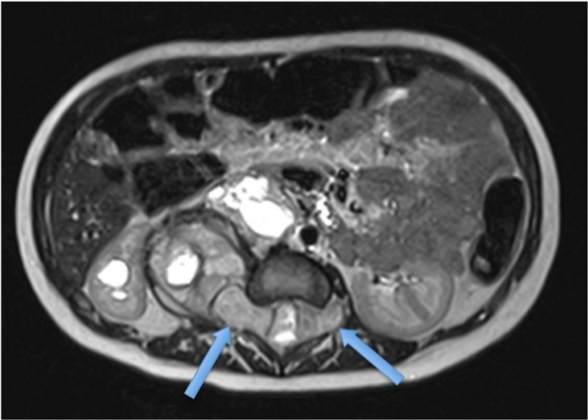
**Axial T2 MR of 3 year old boy showing intraspinal extent of NBL with tumour seen in both neural foramina on this single image (blue arrows).**

**Figure 5:**
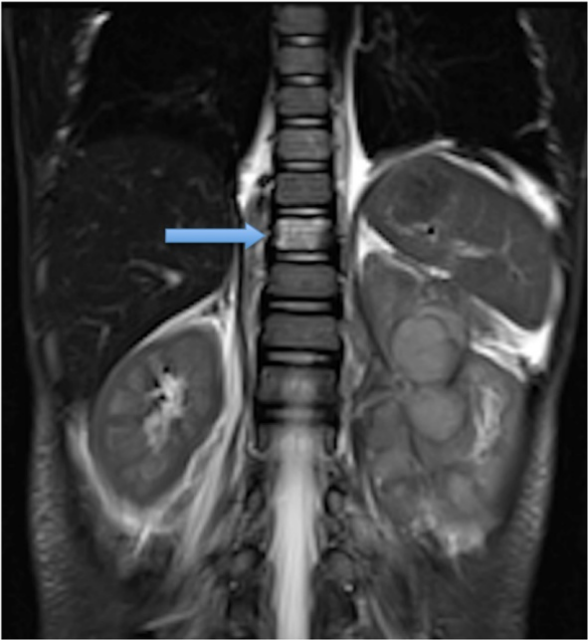
**Coronal T2 MR of a 2 year old boy showing left-sided NBL mass with bone marrow involvement (blue arrow).**

**Figure 6:**
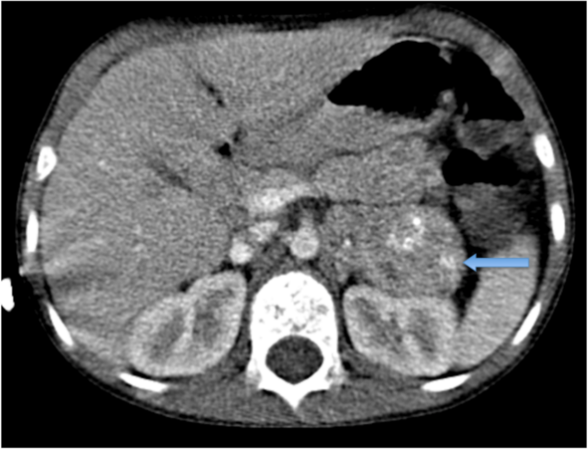
**Axial CT of a 2 year old girl showing a left-sided abdominal NBL with evidence of calcification (blue arrows).**

MRI should now be the cornerstone imaging modality for all primary NBL tumours [Figure 2], whether in the neck, chest, abdomen or pelvis. MRI can easily assess the extent of disease, being superior to CT in assessing metastatic marrow disease, chest wall invasion and spinal canal involvement [Figures 3, 4 & 5]. CT should ideally now, in our opinion, be reserved for pre-operative surgical planning, particularly if there is a surgical preference for CT, when contrast-enhanced images can delineate the vasculature to best effect [1]. With fibrosis and calcification developing after chemotherapy, NBL typically becomes hypointense on T1W and T2W images. The full extent of the mass can be difficult to define on a pre-operative MRI scan and thus make surgical planning more challenging. On a CT study post-chemotherapy, the solid portions of the mass are easier to define than on MRI and the extent of calcification, which increases after treatment and which can be important for the surgeon to appreciate before surgery, is more easily characterised. These features are particularly important when the mass is known to be encasing major vessels. In more localised adrenal or other L1 tumours, pre-operative MRI is preferred over CT as no significant vascular encasement is typically present. On MR at diagnosis, the tumour tends to return low signal on T1-weighted sequences with high signal on T2 [3]. Areas of calcification and haemorrhage can also be detected, the former less reliably [3]. Variable contrast-enhancement may be seen, with the more malignant tumours showing restricted diffusion on diffusion-weighted imaging (DWI). On CT, NBLs are poorly marginated, heterogeneous masses. They can demonstrate extension across the midline and into adjacent body cavities. One of the key defining features is the presence of calcification [Figure 6] seen in 80-90% of CT studies [2]. Despite their size and sometimes aggressive nature, NBLs tend to encase and displace structures rather than invade them [Figure 3]. Vascular invasion is not a classic feature demonstrated on cross-sectional imaging [2].

**Figure 7:**
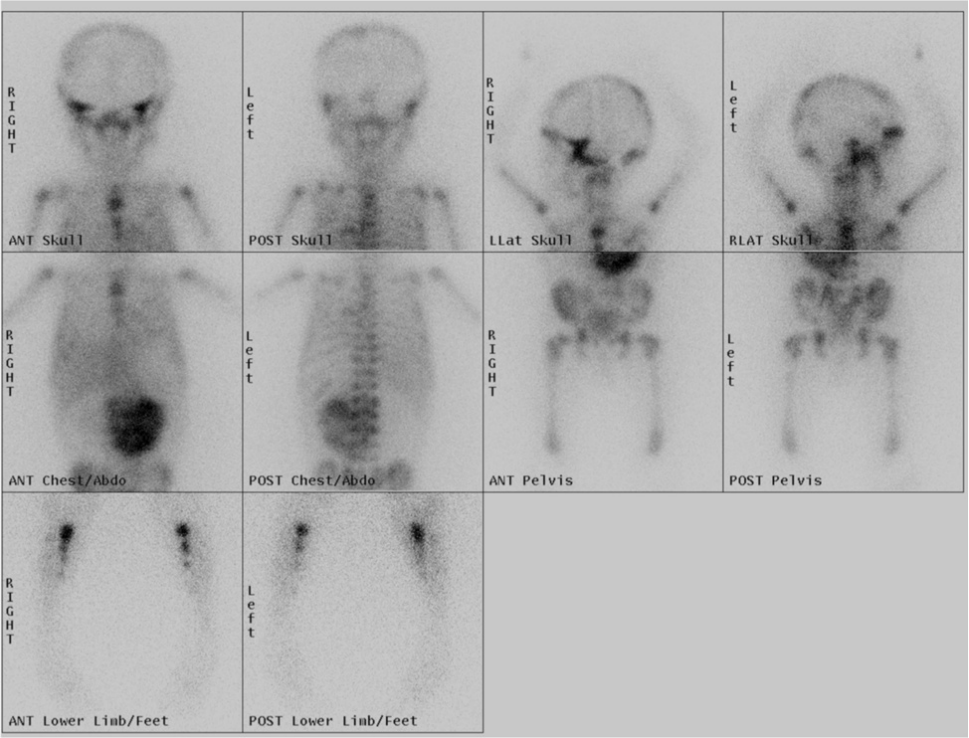
**MIBG scintigraphy showing avid uptake at the site of the abdominal NBL with widespread bony metastatic spread.**

Nuclear medicine studies are utilised in the diagnostic pathway to detect occult disease and assess for distant bony spread. Metaiodobenzylguanidine (MIBG) scintigraphy [Figure 7] is sensitive and specific for NBL because, despite being taken up by other neuroendocrine tumours, these other tumours are extremely rare in the younger paediatric population [3]. Over 90% are MIBG-sensitive, but for those primary tumours that are not MIBG avid, ^99^mTc-diphosphonate bone scintigraphy is currently recommended to look for bony disease [1]. MIBG in its most simple form provides 2D planar information (scintigraphy). It can, however, be acquired in a 3D format with the resultant Single Positron Emission Tomography (SPET or SPECT) images providing more detailed information. Fusing these 3D images with CT can enable tissue differentiation. Studies comparing other functional imaging techniques are currently being undertaken. FDG, fluorodeoxyglucose, is an analogue of glucose that is a positron emitter. The degree of glucose metabolism, and thus uptake of FDG, is higher in tumours such as NBL. FDG combined with CT, PET-CT, allows for more accurate localization of disease. Despite this, there can be the problem of false-positives and false-negatives in non-tumour sites [9]. Studies comparing MIBG to FDG-PET have shown that the former can be sensitive and specific in higher stage disease with FDG-PET being useful in stage 1 and 2 disease or in MIBG non-avid tumours [9]. An emerging PET/CT compound is Ga-68 DOTATATE, which utilizes somatostatin receptor expression in NBLs, in particular sub-type 2 [10,11]. Gallium-68 is a generator produced positron-emitting isotope that is combined with a chelator, DOT, and an octreotide derivative peptide, TATE [10]. The peptide binds to somatostatin receptors and can, therefore, be used for diagnosis and follow-up [10]. DOTATATE is not necessarily limited to use as a diagnostic agent. A study by Gains et al combined the use of Ga-68 DOTATATE for assessment and followed this with Lu-177-DOTATATE for targeted molecular therapy [11]. This showed promising early results as a feasible agent in a select patient cohort [11].

Bone marrow biopsy is also necessary, alongside imaging, to confirm bony disease [6].

#### Staging

The International Neuroblastoma Staging System (INSS) has been used since 1986 to stage NBL [Table 1], however, there was significant worldwide variability in the application of this system, which to some extent is dependent on local protocol and experience [12]. Also, it is a post-surgical staging system and is thus dependent on the expertise of the local surgeon. Assessment of risk pre-surgery was recently deemed to need better defining, with now more reliance placed on pre-operative imaging [8,9]. The International Neuroblastoma Risk Group (INRG) was thus established in 2004 to create a more comprehensive staging system. The INRGSS has 4 simpler stages of disease summarised in Table 2 [13,14]. The INRGSS has not been designed to replace the INSS and centres will likely use both in the management of NBL patients [14]. What INRGSS allows is the pre-surgical assessment of tumours, with imaging making a significant contribution to this. To enable consistent reporting, image defined risk factors (IDRFs) have been identified by the INRG – these describe the relationship between the tumour and adjacent vascular, major airway or nervous system structures, which ideally should not be injured at surgery [6,14].

**Table 1:** **INSS staging system** [11]

**Tumour stage**	**Description**
Stage 1	- Localized tumour with complete gross excision (+/-microscopic residual)
	- Ipsilateral lymph nodes negative for tumour
	- Nodes attached to and removed with tumour may be positive
Stage 2A	- Localized tumour with incomplete gross excision
	- Ipsilateral non-adherent lymph nodes negative for tumour
Stage 2B	- Localized tumour +/- complete gross excision
	- Ipsilateral non-adherent lymph nodes positive for tumour
	- Enlarged contralateral lymph nodes negative for tumour
Stage 3	- Unresectable unilateral tumour infiltrating across midline
	- +/- Regional lymph node involvement
Stage 4	- Any primary tumour with distant spread to lymph nodes, bone, bone marrow, liver, skin and/or other organs (except as defined for stage 4S)
Stage 4S	- Only for infants <1 year
	- Localized primary tumour with disseminated disease limited to skin, liver and/or bone marrow (marrow involvement < 10% on biopsy and MIBG negative marrow)

**Table 2:** **INRGSS** [11,12]

**Tumour stage**	**Description**
L1	- Local disease with no IDRF
L2	- Local disease with one or more IDRF
M	- Distant metastatic disease
MS	- Distant metastatic disease confined to skin, liver and/or bone marrow (<10% involvement) in those under 18 months

#### Image defined risk factors (IDRFs)

The entire number of IDRFs are summarised in Table 3. They can be simplified as major vascular encasement, airway compression or CNS infiltration. Vessel encasement is defined as 50% or greater encirclement of the vessel, with the exception of the renal vasculature where any tumour abutting the renal vessels is regarded as an IDRF [15].

**Table 3:** **Image defined risk factors** [12]

**Risk factor**	**Description**
**Ipsilateral tumour extension within two body compartments**	•Neck-chest
	•Chest-abdomen
	•Abdomen-pelvis
**Neck**	•Tumour encasing carotid and/or vertebral artery and/or internal jugular vein
	•Tumour extending to base of skull
	•Tumour compressing the trachea
**Cervico-thoracic junction**	•Tumour encasing brachial plexus roots
	•Tumour encasing subclavian vessels and/or vertebral and/or carotid artery
	•Tumour compressing the trachea
**Thorax**	•Tumour encasing the aorta and/or major branches
	•Tumour compressing the trachea and/or principal bronchi
	•Lower mediastinal tumour, infiltrating the costo-vertebral junction between T9 and T12
**Thoraco-abdominal**	•Tumour encasing the aorta and/or vena cava
**Abdomen/pelvis**	•Tumour infiltrating the porta hepatis and/or the hepatoduodenal ligament
	•Tumour encasing branches of the superior mesenteric artery at the mesenteric root
	•Tumour encasing the origin of the coeliac axis, and/or of the superior mesenteric artery
	•Tumour invading one or both renal pedicles
	•Tumour encasing the aorta and/or vena cava
	•Tumour encasing the iliac vessels
	•Pelvic tumour crossing the sciatic notch
**Intraspinal tumour extension whatever the location provided that:**	•More than one third of the spinal canal in the axial plane is invaded and/or the perimedullary leptomeningeal spaces are not visible and/or the spinal cord signal is abnormal
**Infiltration of adjacent organs/structures**	•Pericardium
	•Diaphragm
	•Kidney
	•Liver
	•Duodeno-pancreatic block
	•Mesentery
**Conditions to be recorded, but not considered IDRFs**	•Multifocal primary tumours
	•Pleural effusion, with or without malignant cells
	•Ascites, with or without malignant cells

Preliminary evidence suggests absence of IDRFs leads to more complete resection, with the presence of IDRFs resulting in more post-operative morbidity [16]. It is unknown at present if the presence or absence of IDRFs affects overall survival.

#### Management

The COG stratify risk as low, intermediate or high based on prognostic factors and the INSS staging system. Low-risk patients have a 5-year survival rate > 95% with intermediate and high risk group rates of 90-95% and 40-50% respectively [5]. Management strategies include a combination of surgery, chemotherapy and radiotherapy, with additional myeloablative therapy and more recently also immunotherapy for high-risk disease. The clinical approach should involve multidisciplinary discussion following thorough risk-assessment. Low risk patients with local tumour masses should have them surgically resected. This may occur following chemotherapy to try and shrink the mass, ensuring full resection [8]. Intermediate risk patients have chemotherapy followed by surgery [17]. High-risk patients have a more intensive chemotherapy course followed by resection then myeloablative chemotherapy [16]. Radiotherapy directly to the mass is also routinely administered in high-risk tumours post-chemotherapy [8].

### Nephroblastoma (Wilms Tumour)

#### Background

Nephroblastoma is more commonly known as a Wilms tumour after Dr Max Wilms, the German surgeon who first described it in 1899. It is the most common renal malignancy in childhood and accounts overall for 6% of malignancies in children [18]. After hydronephrosis and multicystic dysplastic kidney, it is the most common cause of a renal mass in a child [19]. It typically occurs in childhood with a peak incidence between 3–4 years. It is a little more prevalent in people of African descent.

Wilms tumour is an undifferentiated mesodermal tumour, containing a variable amount of embryonic renal elements (blastema, epithelium and stroma) [20]. There are now two distinct histopathological types based on prognosis – favourable (over 90%) and unfavourable (6-10%). The anaplastic and sarcomatous variants are the unfavourable histologies associated with a poorer outcome [21].

#### Associations and risk factors

**Figure 8:**
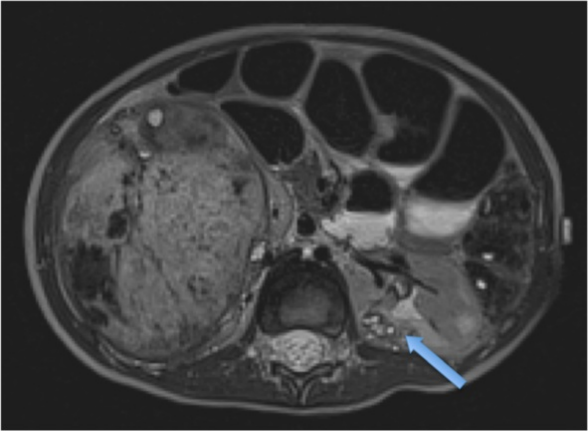
**Axial T2 MR of a 4 year old boy with right-sided Wilms tumour and left-sided nephroblastomatosis (blue arrow).**

Nephroblastomatosis, which consists of immature metanephric tissue (nephrogenic rests), is considered a precursor to Wilms tumour [Figure 8]. The exact risk of development of Wilms tumour on a background of nephroblastomatosis is unclear, however. If a removed kidney containing a Wilms tumour is found to have nephroblastomatosis, there is a 20% chance of developing a Wilms tumour in the contralateral kidney [22].

Syndromes associated with nephroblastomatosis include trisomies 13 and 18, Beckwith-Weidemann (10-20% risk of Wilms; gigantism, macroglossia, omphalocoele and genitourinary abnormalities, associated with an abnormal WT2 gene on 11p15) and Drash syndromes (ambiguous genitalia and progressive renal failure in genotypic males, associated with an abnormal WT1 gene on 11p13).

Other conditions associated with Wilms tumour include hemihypertrophy (WT2 gene), WAGR syndrome (Wilms tumour, aniridia, genitourinary abnormalities and mental retardation, WT1 gene), sporadic non-familial aniridia, neurofibromatosis type 1 and cerebral gigantism (Sotos syndrome) [19,22].

#### Clinical features

Presentation is usually with a large, painless abdominal mass and very little in the way of constitutional symptoms [20]. Up to 10% are discovered incidentally after trauma, 25% have microscopic haematuria and 25% manifest with hypertension secondary to renin production [20].

#### Diagnosis

Plain abdominal radiographs are non-specific for Wilms tumours. If performed, a feature that may be seen is a soft tissue mass displacing loops of bowel.

**Figure 9:**
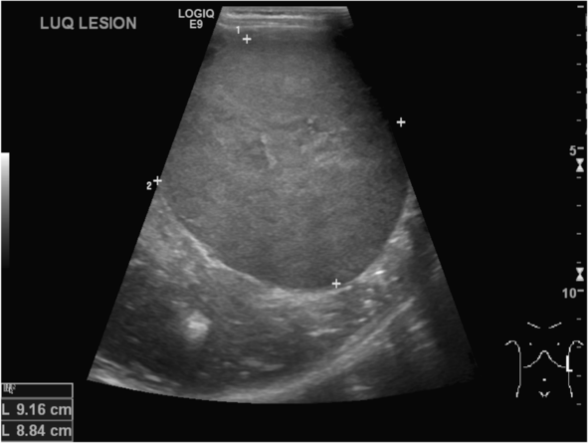
**Ultrasound abdomen of a 4 year old boy with a left-sided Wilms tumour, presenting here unusually as a solid uniform mass.**

Imaging and diagnosis of Wilms tumour generally begins with US [Figure 9], which can evaluate whether the mass is truly intra- or extra-renal and whether it is solid or cystic [23]. It should be noted that often Wilms lesions appear to have large hypoechoic areas due to central necrosis and cyst formation. Hyperechoic areas may represent areas of fat, calcification or haemorrhage. It may also appear less commonly as a solid spherical mass [19]. In contrast to neuroblastoma, vessels are displaced rather than encased as the tumour directly displaces adjacent structures as it grows. Vascular invasion is estimated to occur in approximately 5-10% of cases [24]. US is useful for the assessment of caval patency and IVC tumour thrombus and is the preferred modality for this in our experience. In one North American study, contrast-enhanced CT was more sensitive to disease in these vessels and they report US is not always necessary if a staging CT has already confirmed the presence of thrombus [25]. Renal vein thrombus may be more difficult to evaluate or exclude with US, and CT or MRI tend to be easier to interpret in this setting.

**Figure 10:**
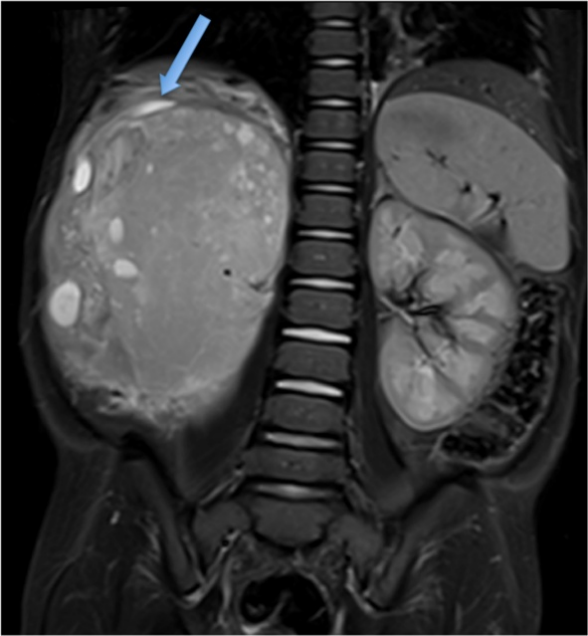
**Coronal T2 fat-saturated MR of 3 year old boy showing a right sided Wilms tumour with the ‘claw sign’ of normal renal tissue (blue arrow) surrounding the tumour.**

**Figure 11:**
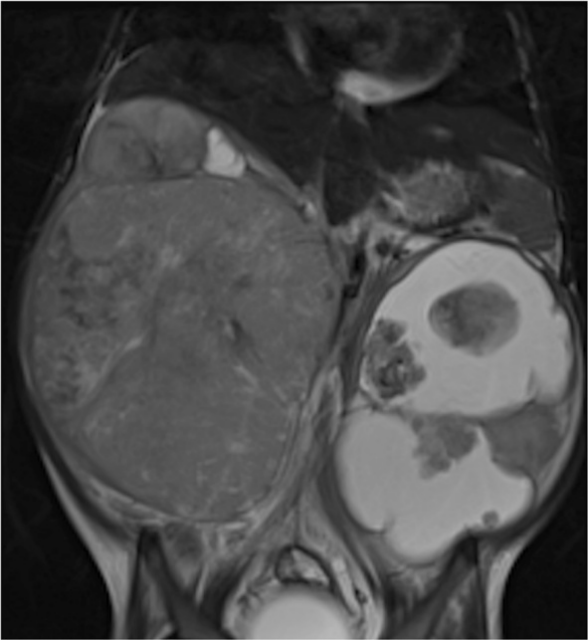
**Coronal T2 MR of a 4 year old girl with bilateral Wilms tumours, more cystic on the left.**

Although yet to be conclusively shown to be superior to CT, the preferred imaging modality at diagnosis is now without doubt contrast-enhanced MRI, in these children with such a favourable long-term outcome. Like CT, MRI can also readily demonstrate the ‘claw sign’ of normal renal tissue around the tumour [Figure 10]. The tumour extent is easily visualised on non-contrast T1W and T2W sequences, but small bilateral tumours and foci of nephroblastomatosis are often best seen after gadolinium administration. Isovolumetric sequences, allowing reconstructions in other orthogonal planes, can be particularly helpful with bilateral disease [Figure 11] when renal preservation surgery is the operative strategy.

MR findings in Wilms tumour are low signal intensity on T1W, with variable/high signal intensity on T2W [26]. Foci of nephroblastomatosis may be small cystic lesions, hyperintense on T2W but sclerotic nephrogenic rests may appear fibrotic, being relatively hypointense on T2W sequences. The non-cystic components of the Wilms mass typically show restricted diffusion on DWI.

**Figure 12:**
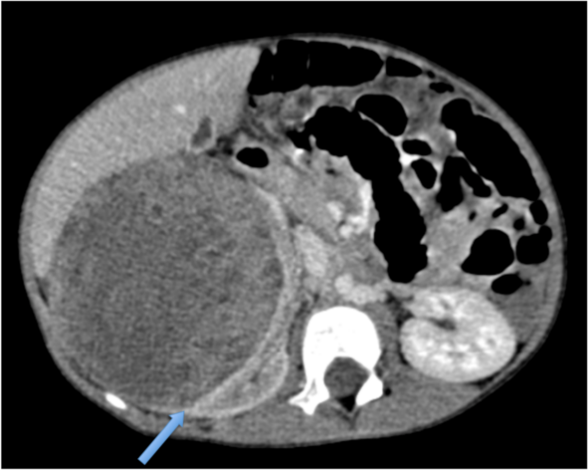
**Axial CT of a 3 year old boy with right-sided Wilms tumour, again demonstrating the ‘claw sign’ (blue arrow).**

Some surgeons may quite reasonably prefer later pre-operative CT prior to surgery, and this would generally be a local preference [Figure 12]. Post-treatment surveillance for bilateral disease should be with MRI and not CT to reduce the radiation burden in these children the vast majority of whom have a good long-term prognosis [27], but some institutions may image post-operatively with CT at the surgeon’s request.

There is continuing uncertainty about the role of pre-operative staging chest CT in the diagnosis of small pulmonary metastases in Wilms tumour. Certainly CT is superior to chest radiography for the detection of small lesions, but these may not always represent metastases. For patients with unilateral Wilms tumour with lung lesions only visible on chest CT, not seen on plain chest radiographs (but deemed metastases and treated as such), their overall survival and event-free survival was no different to those patients whose lung lesions were not deemed metastatic [28]. However, the role of chest CT is not controversial in patients who post-operatively are found to have unfavourable histology or stage III disease, as accurate staging at diagnosis appears to improve overall survival in these patients [28].

PET-CT currently has no role in the initial diagnosis of Wilms tumours as the general prognosis is excellent and radiation exposure should be minimised. In patients who have relapsed, routine PET-CT may be of benefit as their prognosis is more guarded and the best chance of cure is at the first relapse. Accurate staging and discovery of the full extent of metastatic disease would therefore aid survival.

Wilms tumour classically follows the “rule of 10’s”: up to 10% may have unfavourable histology, 10% are bilateral, 10% have vascular invasion, 10% have calcifications on CT and 10% have pulmonary metastases at presentation [19].

#### Staging

Staging of Wilms has been developed by the National Wilms Tumour Study (NWTS) and the current staging system is used by the COG (Table 4). The same post-operative staging system is utilised in European SIOP studies, albeit after courses of pre-operative chemotherapy rather than with upfront surgery as in COG studies. Accurate staging, especially the presence or absence of nodal disease, is vital in Wilms tumour to ensure appropriate management pathways are adhered to [23].

**Table 4:** **Children’s oncology group staging system for Wilms tumour**

**Stage**	**Location**	**Management**
Stage I	Confined to kidney with capsule intact	Complete resection
Stage II	Local spread beyond kidney including renal vein involvement. This includes tumour with local spillage confined to the flank	Complete resection still possible
Stage III	Residual disease confined to the abdomen, including:	Complete resection NOT possible
	a) lymph node involvement	
	b) diffuse peritoneal contamination by growth or spillage	
	c) positive surgical resection margins	
	d) residual non-resected tumour	
Stage IV	Haematogenous metastases	Complete resection NOT possible
Stage V	Bilateral renal involvement	Each kidney should be staged individually

#### Management

A unilateral Wilms tumour is treated with nephrectomy. Neoadjuvant chemotherapy is useful to shrink tumour size prior to surgery, but European (SIOP) and American application of this differs. SIOP prefer pre-operative chemotherapy as surgery is made easier as a consequence and there is less risk of tumour spillage. The result is less stage III disease, thus some patients are down-staged. Radiotherapy is indicated for local stage III, so down-staged patients avoid this and the long-term post-radiotherapy sequelae. There are slightly higher local relapse rates reported in SIOP studies, but these radiotherapy naïve patients do appear to have high salvage rates [29] The American approach is initial surgery and then chemotherapy after post-surgical staging.

In children with bilateral disease, the therapeutic approach and philosophy are very different. Renal preservation surgery becomes paramount. Pre-operative chemotherapy is vital as each kidney is ultimately staged separately. Hemi-nephrectomy, wedge resections and nephron-sparing surgery require accurate pre-operative imaging. The surgical approach in bilateral disease aims to spare any normal renal parenchyma where possible.

Treatment of Wilms tumour is hailed as one of the greatest success stories in modern oncology. Results from the NWTS Group has found overall 10-year survival rates for favourable histology of 96-89% for stages I-III disease (82-49% for unfavourable histology), 81% for stage IV disease (18% for unfavourable histology) and 78% for stage V disease [23].

## Conclusion

Neuroblastoma and Wilms tumour are both relatively common abdominal childhood cancers. Some notable key differences are summarised in Table 5. Considering these factors with new presentations can be helpful with early management strategies, and in initial discussions with a family who have a child with a newly diagnosed malignancy.

**Table 5:** **Summary of key differences between abdominal neuroblastoma and Wilms tumour**

**Parameter**	**Neuroblastoma**	**Wilms tumour**
Age	Younger age group: < 2 years of age commonly	Slightly older age group : peak 3 - 4 years of age
Presentation	Painful abdominal mass	Painless abdominal mass
Calcification	Calcification very common: 80-90%	Calcification uncommon: 10%
Tumour composition	Solid mass lesion, rarely cystic components on US	Often cystic components at US
Tumour margin	Poorly marginated mass that may extend up into chest	Well circumscribed mass - claw sign demonstrating it arises from the kidney
	Adrenal NBL displaces the kidney	
Vessel involvement	Encases vascular structures but does not invade them - elevates the aorta away from the vertebral column	Displaces adjacent structures – invades the vasculature with extension into renal vein/IVC
Metastatic sites	Bone/bone marrow (common)	Lung (common)
	Liver	Liver
	Lung/pleura	Local lymph nodes

## Abbreviations

NBL: Neuroblastoma
pNET: Primitive neuroectodermal tumour
COG: North American Children’s Oncology Group
SIOPEN: International Society of Paediatric Oncology Europe Neuroblastoma
VIP: Vasoactive intestinal peptide
US: Ultrasound
DWI: Diffusion weighted imaging
MIBG: Metaiodobenzylguanidine
INRC: International Neuroblastoma Response Criteria
INSS: International Neuroblastoma Staging System
INRG: International Neuroblastoma Risk Group
INRGSS: International Neuroblastoma Risk Group Staging System
IDRF: Imaging Defined Risk Factors
NWTS: National Wilms Tumour Study
